# Can coefficient of variation of time-domain analysis be valuable for detecting cardiovascular autonomic neuropathy in young patients with type 1 diabetes: a case control study

**DOI:** 10.1186/s12872-016-0467-0

**Published:** 2017-01-19

**Authors:** Dovile Razanskaite-Virbickiene, Evalda Danyte, Giedre Mockeviciene, Rimante Dobrovolskiene, Rasa Verkauskiene, Rimantas Zalinkevicius

**Affiliations:** 1 Department of Endocrinology Medical Academy, Lithuanian University of Health Sciences, LT-44307 Kaunas, Lithuania; 2 Institute of Endocrinology, Medical Academy, Lithuanian University of Health Sciences, LT-44307 Kaunas, Lithuania

**Keywords:** Type 1 diabetes, Heart rate variability, Spectral analysis, Cardiovascular autonomic neuropathy

## Abstract

**Background:**

Cardiovascular autonomic neuropathy (CAN) increases morbidity and mortality in diabetes through association with a high risk of cardiac arrhythmias and sudden death, possibly related to silent myocardial ischemia. During the sub-clinical stage, CAN can be detected through reduction in heart rate variability (HRV). The aim of our study was to estimate if the time and frequency-domain analysis can be valuable for detecting CAN in young patients with type 1 diabetes mellitus (T1DM).

**Methods:**

For this case control study of evaluation of cardiovascular autonomic function the 15–25 years age group of patients with duration of T1DM more than 9 years (*n* = 208, 89 males and 119 females) were selected. 67 patients with confirmed CAN were assigned to the “case group” and 141 patients without CAN served as a control group, the duration of T1DM was similar (15.07 ± 4.89 years vs.13.66 ± 4.02 years; *p* = 0.06) in both groups. Cardiovascular autonomic reflex tests and time and frequency domains analysis of HRV were performed for all subjects.

**Results:**

Time domain measures were significantly lower in CAN group compared with control (*p* < 0.05). R-R max / R-R min ratio and coefficient of variation (CV) were the lowest during deep breathing among T1DM patients with CAN. Receivers operating characteristic (ROC) curves were constructed to compare the accuracies of the parameters of time-domain analysis for diagnosing CAN. We estimated a more reliable cut-off value of parameters of time-domain. The CV values in supine position <1.65, reflected sensitivity 94.3%, specificity 91.5%. The CV values during deep breathing <1.45 reflected sensitivity 97.3%, specificity 96.2%. The CV values in standing position <1.50 reflected sensitivity 96.2%, specificity 93.0%. The most valuable CV was during deep breathing (AUC 0.899). The results of frequency-domain (spectral analysis) analysis showed significant decrease in LF power and LFPA, HF Power and HFPA, total power among subjects with CAN than compared with subjects without CAN (*p* < 0.05).

**Conclusions:**

Time and frequency domain analysis of HRV permits a more accurate evaluation of cardiovascular autonomic function, providing more information about sympathetic and parasympathetic activity. The coefficient of variation (time-domain analysis) especially during deep breathing could be valuable for detecting CAN.

## Background

Cardiovascular autonomic neuropathy (CAN) is one of the most clinically significant and overlooked of all serious complications of diabetes mellitus (DM) [1]. CAN occurs when peripheral autonomic fibres (sympathetic and parasympathetic) of the cardiovascular system are affected, resulting in abnormalities in heart rate (HR) control and vascular dynamics [2, 3]. CAN is associated with a high risk of cardiac arrhythmias and sudden death, possibly related to silent myocardial ischemia, and significantly increases morbidity and mortality risk in diabetes [4]. CAN may have greater predictive power than traditional risk factors for cardiovascular events [5], increasing risk of premature death in type 1 diabetes mellitus (T1DM) with CAN fourfold [6].

CAN might be subclinical for several years until the patient develops resting tachycardia, exercise intolerance, postural hypotension, cardiac dysfunction and diabetic cardiomyopathy [2]*.* The time scale for CAN progression from subclinical to clinical stage is unknown. During the sub-clinical stage, CAN could be detected through abnormalities (reduction) in heart rate variability (HRV) [7–9]. Cardiovascular autonomic reflex tests (CARTs) are simple non-invasive tests to measure cardiac autonomic function based on the heart rate (HR) and blood pressure response to certain physiological manoeuvres. In healthy individuals, the beat-to-beat variability with aspiration is predominantly affected by the direct sympathetic and parasympathetic activity [9]. CARTs are the gold standard in clinical autonomic testing, they have good sensitivity, specificity, and reproducibility and are non-invasive, safe, well-standardized [10, 11].

Newer methods for detecting CAN are more sensitive, and abnormalities in frequency and time domains of HRV analysis may be detected before the development of abnormalities in CARTs [12–14]. HRV can be assessed either by calculation of indices based on statistical analysis of R-R intervals (time-domain analysis) or by spectral analysis (frequency-domain analysis) [10]. Power spectral analysis is a modern technology, which uses a mathematical algorithm (fast Fourier transform) to turn a complex biological signal, such as HRV (result of the sympathovagal balance in the sinus node), into its causing components, presenting them according to the frequency with which they alter the HR [15]. The power spectrum of HRV has been shown to consist of three major peaks [2]: very low-frequency component is related to fluctuations in the vasomotor tonus linked to thermoregulation and sweating (sympathetic control); low-frequency component associated with the baroreflex (sympathetic control with n. vagus modulation); high-frequency component which is related to sinus arrhythmia (parasympathetic control).

Studies have shown that HRV abnormalities can even be present at the time of diabetes diagnosis [13]. Early detection and good glycaemic control have been proven to prevent or delay adverse outcomes associated with diabetes complications [16, 17]. Therefore preventive screening is required in order to identify CAN in its earliest stages and particularly for those, who has diabetes from childhood. There is some evidence that puberty is a threshold for the development of AN, because changes during puberty may accelerate microvascular complications of diabetes [18, 19].

The aim of our study was to estimate if the time and frequency-domain analysis can be valuable for detecting CAN in young patients with T1DM.

## Methods

### Study population

A case control study was conducted in a single research centre as a part of joint Lithuanian – Swiss project “Genetic Diabetes in Lithuania”. The principal aim of the project was to screen for autoimmune antibodies and in order to select patients for the searching monogenic diabetes and to compare the antibody positive with the antibody negative population of the diabetes registry (<25 years of age). The total project cohort consisted of 1209 subjects covering all paediatric patients (<18 years, *n* = 860), and part of adult patients younger than 25 years (*n* = 349) diagnosed with T1DM in Lithuania. All patients had a physician diagnosis of T1DM between March 1990 and March 2015. In patients at the age less than 15 years insulin-dependent diabetes was diagnosed according to DIAMOND criteria [20]: diagnosis confirmed by physician, age at the onset less than 15 years, the date of the onset coincide with the day of the first insulin injection, the person is inhabitant of Lithuania. In adult persons insulin-dependent diabetes was diagnosed according to criteria [21]: diagnosis confirmed by physician, age at the onset 15–39 years, the date of the onset coincide with the day of the first insulin injection, the person is inhabitant of Lithuania, ketones present in urine on the time of diagnosis of diabetes. Patients were identified from Lithuanian national diabetes data base and invited to participate in the study on their visit to the family doctor and/or endocrinologist, a paediatric endocrinologist, meetings of diabetes societies, as well as by post, e-mails and phone calls.

For this case control study of evaluation of cardiovascular autonomic function we selected the 15–25 years age group of patients with duration of T1DM more than 9 years (*n* = 208, 89 males and 119 females). Sixty-seven patients with confirmed CAN were assigned to the case group and 141 patients without CAN served as a control group in this analysis, the duration of T1DM was similar (15.07 ± 4.89 years vs.13.66 ± 4.02 years; *p* = 0.06) in both groups.

The study was approved by local ethical committee (No. BE–2-5/2013) and written informed consent was obtained from all study participants and their parents or official care-givers. The investigation was carried out in accordance with the Declaration of Helsinki.

### Materials

An electrocardiogram (ECG) of CARTs was recorded using AT-101 CARDIOVIT device (Schiller, Swiss, Software analysis SEMA-200). Before assessment, participants avoided alcohol, smoking, coffee and were at least 2 h after a light meal. The absence of marked hyperglycaemia or hypoglycaemia was confirmed measuring capillary blood glucose. Participants were examined in the supine position after 10–15 min of rest in the morning (from 8:00 to 11:00 a.m.).

*A baseline recording* was obtained during normal breathing in a quiet room.

*Deep breathing test:* ECG recording was obtained over 2 min. during deep breathing at a frequency of 6 breaths per min. (5 s in and 5 s out). The expiration vs. inspiration ratio (E vs. I ratio) was determined by the ratio between the longest and shortest R-R intervals obtained during the expiration and inspiration cycles, respectively, and the highest E:I ratio was considered.

*Heart rate response to active standing (orthostatic):* After lying in the supine position for at least 10 min., the subject stand up quickly. The 30 vs. 15 ratios were determined by the ratio between the longest and shortest RR intervals (maximum bradycardia / maximum tachycardia).

*Orthostatic hypotension test:* After at least 10 min. of supine rest, the blood pressure was measured at baseline and after 3 min. of standing. Drops in the systolic blood pressure (SBP) higher or equal to 20 mmHg or in the diastolic blood pressure (DBP) higher or equal to 10 mmHg were considered abnormal.

*Diastolic blood pressure response to isometric exercise* (hand grip using dynamometer): The subject squeezes a handgrip dynamometer to establish a maximum. Grip is then squeezed at 30% maximum for 5 min. The abnormal response for diastolic blood pressure was a rise of less than 10 mmHg in the other arm [22].

CAN was diagnosed based on at least two abnormal cardiovascular autonomic reflex test results.

ECG of time and frequency-domain tests was recorded with Neuropack MEB-9400 device (NIHON KOHDEN, Japan) with special software QP-948 BK for exploration of autonomic nervous system (for analysis of HRV indices). The device takes into account the normal hearts beats, derives the statistical parameters of the normal R-R intervals (NN) and computes time and frequency domain HRV indices. To avoid noise and artefact, the participants and the electrode junction box were placed more than 1 m away from the system. The participants were instructed to remain at rest, to avoid movements and conversations during data collection. Three surface electrodes were placed on the chest to obtain an electrocardiogram tracing. We have possibility using software QP-948 BK to eliminate ectopic beats, noises and artefacts after visual detection. Short-term recordings (up to 5 min. in the resting state and up to 2 min. during deep breathing and standing) yield up to two peaks in low and high frequency ranges.

Time-domain parameters were analysed: the ratio of maximum R-R interval / minimum R-R interval (R-R max / R-R min), the standard deviation of R-R interval (SD), the percentage of differences between adjacent RR intervals > 50 ms (pNN50) and coefficient of variation of R-R interval (CV) (CV = SD/mean R-R interval*100). Frequency-domain parameters were analysed: LF range (0.04–0.15-Hz), LF Power - power of the highest peak in the lower frequency range, LFPA - total power of the lower frequency range; HF range (0.15–0.4 Hz), HF Power - power of the highest peak in the higher frequency range, HFPA - total power of the higher frequency range; LF-to-HF ratio; LFPA-to-HFPA ratio; and total power (TP) of the spectrum (0.003–0.4 Hz). The spectral analysis was calculated using a mathematical Fast Fourier Transform algorithm.

Standardised questionnaires were completed to obtain information on demographic data, clinical events, medications and life style.

*Laboratory tests:* The following laboratory tests were performed: glycated haemoglobin (HbA1c), total cholesterol, high density lipoprotein (HDL), low density lipoprotein (LDL), triglycerides (TG).

### Statistical data analysis

The statistical analysis was conducted using the SPSS 22.0 statistics software package. The mean, standard deviation (SD) and the 95% confidence interval (CI) are indicated for quantitative variables; and value rates as well as relative rates in percent are indicated for qualitative variables.

In order to determine the sample size, the force β = 0.8 and the probability value α =0.05 were chosen.

The Student t test was used to check the average equality hypothesis in case of normal data distribution and the Mann-Whitney test in case of non-normal data distribution.

The independence of qualitative variables was tested using the chi-square χ^2^ test; and if the number of monitored patients was low, the Fisher exact criterion was applied.

The areas under the Receiver Operating Characteristics (ROC) curves were calculated. Possible dependent variable values and their specificity and sensitivity were estimated.

The significance level 0.05 was chosen to test statistical hypotheses.

## Results

The 208 patients with T1DM (89 males and 119 females) were included in the study. The characteristics of patients are summarized according CAN in Table 1. There were no significant differences in mean diabetes duration and gender distribution among study subjects with and without CAN (cases vs. controls). SBP and HDL were similar among groups (*p* > 0.05). However, DBP and HR were significantly higher among case group compared with control group. T1DM patients with CAN (cases) were more likely to have a worse metabolic profile: higher HbA1c (*p* = 0.002), total cholesterol (*p* < 0.001), LDL (*p* = 0.003), TG (*p* = 0.005).

**Table 1:** Characteristics of type 1 diabetes according to the presence of CAN

Variable	T1DM with CAN (case patients)	T1DM without CAN (control patients)	p
N	67	141	
Gender (M/F)	29/38	60/81	0.23
Age (years)	20.04 ± 4.91	18.82 ± 3.86	<0.01
Diabetes duration (years)	15.07 ± 4.89	13.66 ± 4.02	0.06
SBP (mmHg)	124.58 ± 10.12	118.12 ± 8.62	0.07
DBP (mmHg)	77.53 ± 8.72	74.58 ± 6.23	<0.01
HR (bpm)	81.37 ± 9.93	73.70 ± 8.65	<0.01
HbA1c (%)	9.46 ± 1.63	8.77 ± 1.80	0.002
Total cholesterol (mmol/l)	5.64 ± 1.23	4.88 ± 1.03	<0.001
HDL (mmol/l)	1.26 ± 0.37	1.30 ± .0.25	0.16
LDL (mmol/l)	3.11 ± 0.89	2.79 ± 0.78	0.003
Triglyceride (mmol/l)	1.24 ± 0.65	1.06 ± 0.42	0.005

Note: Data are means (standard deviation) unless otherwise indicated

CARTs measures were analyzed and compared between young T1DM patients with CAN and without CAN. Results of analysis are presented in Table 2. All CARTs measures were significantly lower in case group than in control (*p* < 0.05).

**Table 2:** Comparison of CARTs results between case and control groups

	Mean	−95 CI	+95 CI
R-R index of deep breathing (E:I)			
Case group	1.13	1.09	1.17
Control group	1.34	1.31	1.36
p	<0.001		
R-R index of active standing (30:15)			
Case group	1.14	1.10	1.17
Control group	1.26	1.23	1.28
p	<0.001		
SBP ∆ (orthostaic hypotension test)			
Case group	−6.01	−7.55	−4.44
Control group	3.63	1.16	5.10
p	0.005		
DBP ∆ (hand grip using dynamometer)			
Case group	8.30	7.64	8.36
Control group	16.47	15.56	16.51
p	0.002		

Note: ±95 proc. *CI* confidence intervals

We analysed if gender influenced the abnormal changes of CARTs among type1 diabetic patients with CAN (Table 3). We have found no significant differences in abnormal CARTs measures between males and females with T1DM and CAN (*p* > 0.05).

**Table 3:** Statistical indicators of abnormal changes of CARTs according to gender among type 1 diabetic youth with CAN

	Mean	−95 CI	+95 CI
Index of deep breathing (E:I) ≤1.1			
Male	1.01	1.01	1.02
Female	1.02	1.01	1.03
p	0.82		
Index of orthostatic (30:15) ≤1.1			
Male	1.04	1.02	1.06
Female	1.04	1.03	1.06
p	0.76		
SBP Δ (orthostatic hypotension) ≤ −20 mmHg			
Male	−22.00	−27.55	−16.44
Female	−23.63	−28.16	−19.10
p	0.84		
DBP Δ (hand grip) ≤ −10 mmHg			
Male	−12.30	−14.65	−9.96
Female	−13.47	−15.76	−11.18
p	0.43		

Note: ±95 proc. *CI* confidence intervals

Time domain measures of HRV were computed for R-R intervals in supine position, deep breathing and standing position both in case and control groups. The results of time domain analysis are summarized in Table 4. All measures were significantly lower in case group when compared with control group (*p* < 0.05). R-R max / R-R min ratio and CV were the lowest during deep breathing among T1DM patients with CAN. However, T1DM patients without CAN showed the lowest R-R max / R-R min ratio and CV in supine position.

**Table 4:** Differences of HRV parameters (time-domain method) between type 1 diabetic patients with and without CAN

Variable	T1DM with CAN	T1DM without CAN	p
Supine position			
R-R max / R-R min	1.12 ± 0.07	1.28 ± 0.18	<0.001
SDNN (ms)	16.69 ± 8.50	45.16 ± 25.92	<0.001
pNN50 (%)	0.95 ± 0.65	20.10 ± 2.56	<0.001
CV (%)	2.27 ± 1.07	4.63 ± 2.38	<0.0001
Deep breathing			
R-R max / R-R min	1.1 ± 0.05	1.36 ± 0.24	<0.001
SDNN (ms)	31.87 ± 13.68	41.13 ± 16.24	0.02
pNN50 (%)	1.60 ± 0.87	26.63 ± 2.38	<0.001
CV (%)	2.09 ± 0.98	6.72 ± 4.27	<0.0001
Standing position			
R-R max / R-R min	1.14 ± 0.18	1.38 ± 0.27	<0.001
SDNN (ms)	24.85 ± 5.19	56.12 ± 4.44	<0.001
pNN50 (%)	0.71 ± 0.50	12.18 ± 6.59	<0.001
CV (%)	2.59 ± 1.65	6.37 ± 4.05	<0.0001

Note: Data are means (standard deviation)

The results of frequency-domain (spectral analysis) analysis are presented in Table 5. LF power and LFPA (showing sympathetic control with n. vagus modulation) were significantly lower among T1DM patients with CAN (cases) than compared with T1DM patients without CAN (controls) (*p* < 0.05). Also HF Power and HFPA (showing parasympathetic control only) were significantly lower among case group than compared with control group (*p* < 0.05). HFPA was lower than LFPA among both groups. Total power (corresponds to the sum of all spectral bands) was significantly lower among subjects with CAN than in subjects without CAN (*p* < 0.05). There were no significant differences in LF/HF and LFPA/HFPA (reflects global sympathetic - parasympathetic balance) among groups (*p* > 0.05).

**Table 5:** Differences of HRV parameters (frequency-domain method) between type 1 diabetic patients with and without CAN

Variable	T1DM with CAN	T1DM without CAN	p
Spectral analysis			
LF Power (ms^2^)	2.26 ± 0.28	5.79 ± 1.24	<0.01
LFPA (ms^2^)	10.13 ± 1.28	26.78 ± 4.78	<0.001
HF Power (ms^2^)	1.47 ± 0.22	3.97 ± 0.64	<0.001
HFPA (ms^2^)	7.39 ± 1.11	20.07 ± 2.83	<0.0001
LF/HF (%)	32.39 ± 1.95	37.64 ± 2.69	0.11
LFPA/HFPA (%)	175.01 ± 26.30	142.25 ± 14.49	0.30
T Power (ms^2^)	29.36 ± 3.19	70.48 ± 9.17	<0.001

Note: Data are means (standard deviation)

**Fig. 1:**
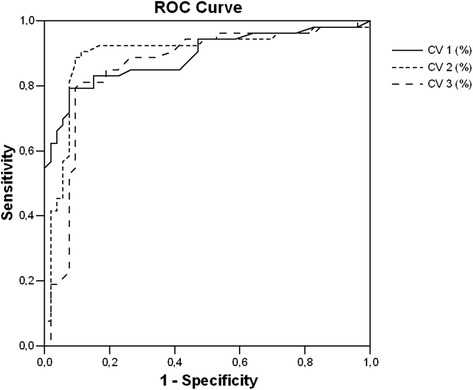
ROC curve and corresponding AUC of the CV in distinguishing cases from controls are presented. Note: CV 1 – in supine position, CV 2 – during deep breathing, CV 3 – in standing position

Receivers operating characteristic (ROC) curves were constructed to compare the accuracies of the parameters of time-domain analysis for diagnosing CAN (Table 6, Fig. 1). We estimated a more reliable cut-off value of parameters of time-domain. The CV values in supine position <1.65 reflected sensitivity 94.3%, specificity 91.5%. The CV values during deep breathing <1.45 reflected sensitivity 97.3%, specificity 96.2%. The CV values in standing position <1.50 reflected sensitivity 96.2%, specificity 93.0%. So the most valuable CV was during deep breathing (AUC 0.899).

**Table 6:** Summary ROC plot of sensitivity versus specificity of the coefficients of variation for CAN

Variable	Value	AUC	±95% CI	Sn (%)	Sp (%)
CV1 in supine position	<1.65	0.890 ± 0.033	0.825–0.955	94.3	91.5
CV2 in deep breathing	<1.45	0.899 ± 0.035	0.831–0.967	97.3	96.2
CV3 in standing position	<1.50	0.862 ± 0.039	0.785–0.939	96.2	93.0

Note: *AUC* area under the curve, *Sn* sensitivity, *Sp* specificity

## Discussion

The results of our study showed the reduction of overall HRV in young T1DM patients with confirmed CAN, when compared with patients with the similar duration of disease and glycaemic control, but without CAN. HRV in deep breathing under parasympathetic control was most often impaired in CAN. This study has demonstrated the importance of the CV for diagnosing CAN. Spectral analysis confirmed the significant decreases in parameters of HF and LF ranges. Our study results confirmed that a decrease in HRV is an early sign of CAN.

The reported prevalence of CAN varies, depending on the type and number of tests performed, different criteria used to diagnosed autonomic dysfunction and also the patient cohort studied [23]. Ziegler et al., evaluated a large cohort of patients (647 T1DM and 524 T2DM) and found that 25.3% of patients with T1DM and 34.3% of patients with T2DM had abnormal findings in more than two of six autonomic function tests [24]. CAN could be detected in about 7% of T1DM at the time of initial diagnosis, and it is estimated that the risk for developing CAN increases annually by approximately 6% [25]. The improvement of glycaemic control could reduce the prevalence and the progression of CAN. Autonomic failure was confirmed in 12.3% of people with T1DM in French study in 2010 [26]. Moreover, there are evidences that autonomic dysfunction may be accelerated by puberty suggest the screening of adolescents and young adults for CAN [27].

HbA1c, hypertension, age, hypertrigliceridaemia, dyslipidaemia, gender (female), diabetic symmetric peripheral neuropathy, albuminuria, retinopathy, and exposure to hyperglycemias are risk factors for developing CAN among T1DM [28]. Subjects with confirmed CAN had significant higher DBP and worse lipid profile (*p* < 0.05) in our study. The association of CAN with other diabetes microangiopathic complications should lead to consider CAN as an indicator of them [26]. Antony et al. examined the relationship between 24-h blood pressure measurements, urinary albumin excretion rates, and autonomic neuropathy in adolescents with T1DM. They concluded that higher 24-h BP values and evidence of subclinical signs of autonomic neuropathy are present before persistent microalbuminuria develops and may have important implications for timing the introduction of early treatments designed to prevent or retard the microvascular complications in T1DM adolescents [29]*.*

It is important to diagnose CAN in its subclinical (early) stage. Lack of HRV during deep breathing or exercise is a sign of autonomic nerve impairment. Reduced HRV is the earliest indicator of CAN [30]. Resting HR of 100–130 bpm are a manifestation of later stages of the disease and reflect the relative increase in the sympathetic tone associated with n. vagus impairment [31]. Later develops combined parasympathetic and sympathetic damage, HR returns toward normal but still remains elevated [10]. A fixed HR that is unresponsive to moderate exercise, stress, or sleep indicates almost complete cardiac denervation [31]. In our study young T1DM patients with CAN had significant higher resting HR when compared with control group (*p* < 0.05). Vanderlei et al. have tried to verify possible associations between HRV indices and physical activity, body composition, and metabolic and cardiovascular parameters in individuals with T1DM. They have found that for young patients with T1DM, increases in at-rest HR values are associated with reduced parasympathetic activity and global HRV, whereas higher waist-to-hip ratio values are related to lower parasympathetic activity, both independent of age and gender [32].

CAN is still widely under-diagnosed, despite its high prevalence and impact on morbidity and mortality. Despite the existing guidelines for the diagnosis of CAN, there is no widespread standardized method to CAN testing [33]. Autonomic symptoms are nonspecific and do not permit diagnosis of CAN. CARTs are the gold standard in clinical autonomic testing [10]. The most widely used tests assessing cardiac parasympathetic function are based on HR response to deep breathing (expiration/inspiration ratio), active standing (maximum/minimum 30:15 ratio) and a Valsalva manoeuvre. HR to deep breathing has the greatest specificity (80%). However, CARTs have some contraindications and they require a very good cooperation of patients. A Valsalva manoeuvre must not be performed in patients with proliferative retinopathy. That’s why we didn’t included it in our study. The sympathetic function is assessed by measuring the blood pressure response to orthostatic change, hand grip using dynamometer and a Valsalva manoeuvre. In our study all CARTs measures of T1DM young patients with CAN were significantly lower than those without CAN (*p* < 0.05).

New methods for detecting CAN offer to assess HRV either by calculation of indices based on statistical analysis of R-R intervals or by spectral analysis [10]. They are easily performed and do not require cooperation of patients. In SEARCH CVD study HRV parameters were measured in 354 young patients with T1DM (mean age 18.8 years, diabetes duration 9.8 years, and mean A1C 8.9%) and 176 young persons without diabetes (mean age 19.2 years). Youth with T1DM had reduced overall HRV and markers of parasympathetic loss (reduced HF power) with sympathetic override (increased LF power) when compared with control subjects. So, a conclusion was that diabetic youth with signs of early CAN have reduced overall HRV and parasympathetic loss with sympathetic override [34]. Jovarka et al. have tested the hypothesis that cardiovascular regulation is abnormal in young patients with T1DM. In young patients with T1DM significant reduction of spectral power in HF band of the HRV was found, whereas no significant difference between DM group and control group was observed in LF band of blood pressure variability. They suggested that abnormalities in cardiac parasympathetic regulation precede impairment of blood vessels sympathetic control in young diabetics [35]. The measures of time domain analysis of our study were significantly lower among T1DM youth with CAN when compared with control group (*p* < 0.05). Also R-R max / R-R min ratio and CV were the lowest during deep breathing among T1DM youth with CAN (showing decrease in parasympathetic activity). We analysed sensitivity and specificity of the coefficient of variation (parameter of time-domain analysis) for diagnosing CAN and have found that the most valuable CV was during deep breathing (values <1.45 reflected sensitivity 97.3%, specificity 96.2%). The results of spectral analysis of our study confirmed the significant decrease in HF, LF and total power (showing decrease in parasympathetic and sympathetic activity) among patients with CAN. Also we have found that LFPA and HFPA were significantly lower among cases than compared with control group, but these indices are not so valuable from mathematical point of view. Power Spectral Analysis and HRV have been employed in trials for the detection of autonomic neuropathy in patients with Charcot’s disease. Similarly to Charcot’s arthropathy, patients with recurrent vascular neuropathic ulcers appear to share analogous cardiac autonomic dysfunction [9]. Hikita et al. [36] demonstrated that HRV is reduced in diabetic patients with silent ischemia when compared with non-diabetic patients with silent or painful ischemia in 24-h ambulatory electrocardiographic recordings. Katz et al. showed that a simple test that measured 1-min HRV during deep breathing was a good predictor of all-cause mortality for 185 patients (17.8% with diabetes) after a first MI [10].

Various studies are trying to create more useful tool for the early detection of CAN in patients with T1DM [37, 38]. Jovarka et al. [38] have analysed if a new HRV complexity measure, the Point Correlation Dimension (PD2i), could provide diagnostic information regarding early subclinical autonomic dysfunction in T1DM. The R-R intervals were measured over 1 h with a telemetric ECG system. They have found that PD2i was able to detect ANS dysfunction with *p* = 0.0006, similar to the best discriminating MSE scale, with *p* = 0.0002.

So, the significance of CAN has not been fully appreciated and remains among the least understood and least frequently diagnosed diabetes complications, despite its significant negative impact on survival and quality of life [2]. According ADA recommendations [39], diagnostic tests of CAN should be performed for T1DM: 5 years after the diagnosis; before planning a program of moderate-to-high-intensity physical exercise; with a history of poor glycaemic control, high cardiovascular risk and microangiopathic complications.

One of the weaknesses of our case control study is a narrow range of T1DM duration (more than 9 years). We intend to evaluate CAN among T1DM with disease duration more than 5 years or even from the diabetes onset. The other weakness is that HRV was evaluated from a short length of recording (5 min.). Another weak point - we had only one HbA1c measurement not reflecting longstanding glycaemic control. The major strength of our study is the young T1DM population without concomitant medications and comorbidities interfering results of tests, homogeneous according diabetes duration. The study used various parameters of time and frequency domains analysis for the analysis of HRV.

## Conclusions

Time and frequency domain analysis of HRV permits a more accurate evaluation of cardiovascular autonomic function, providing more information about sympathetic and parasympathetic activity. The coefficient of variation (time-domain analysis) especially during deep breathing could be valuable for detecting CAN.

## Abbreviations

CAN: Cardiovascular autonomic neuropathy
CARTs: Cardiovascular autonomic reflex tests
CV: Coefficient of variation
DM: Diabetes mellitus
HF: Higher frequency
HR: Heart rate
HRV: Heart rate variability
LF: Lower frequency
T1DM: Type 1 diabetes mellitus
TP: Total power
